# Influence of a Medium-Impact Exercise Program on Health-Related Quality of Life and Cardiorespiratory Fitness in Females with Subclinical Hypothyroidism: An Open-Label Pilot Study

**DOI:** 10.1155/2013/592801

**Published:** 2013-12-31

**Authors:** Andrea Garces-Arteaga, Nataly Nieto-Garcia, Freddy Suarez-Sanchez, Héctor Reynaldo Triana-Reina, Robinson Ramírez-Vélez

**Affiliations:** ^1^Departamento de Educación Física y Deporte, Universidad del Valle, Meléndez Cali, Colombia; ^2^Grupo GICAEDS, Facultad de Cultura Física, Deporte y Recreación, Universidad Santo Tomás, Carrera 9 N° 51-23, Bogotá, DC, Colombia

## Abstract

*Objective.* To examine the influence of a medium-impact exercise program (MIEP) on health-related quality of life (HRQoL) and cardiorespiratory fitness (VO_2max_) in females with subclinical hypothyroidism (sHT). *Materials and Methods.* We selected 17 sedentary women with sHT (mean age: 43.1 (standard deviation: 9.7) years). Participants carried out an MIEP consisting of 3 weekly sessions of 60 minutes during 12 weeks. Before and after the exercise program HRQoL was assessed by the SF-12v2 questionnaire, and VO_2max_ was evaluated by Rockport walk test. *Results.* After the 12-week intervention, the participants that performed an MIEP showed improvements in HRQoL in most domains, particularly the vitality domain by 7 points, the social functioning domain by 10 points, the mental health domain by 7 points, and the mental component summary by 7 points. One of the four domains within the physical component summary (general health domain) showed significant effect of the exercise intervention: 6 points. Moreover, the participants that performed exercise showed a higher VO_2max_ (28%; *P* < 0.01). *Conclusion.* After 12 weeks of medium-impact exercise program, there were remarkable improvements in HRQoL in most domains. Moreover, this exercise program proved to have a positive influence on cardiorespiratory fitness.

## 1. Introduction

Prevalence of subclinical hypothyroidism (sHT), defined as elevated thyroid stimulating hormone (TSH) with free thyroxine (fT4) in the normal range, increases with age affecting about 6% of individuals aged 70 to 79 years and 10% of those aged 80 or older [1, 2]. Patients with sHT are associated with increased prevalence of atherosclerotic lesions and cardiovascular events [3, 4]. Besides, thyroid hormone deficiency may also interfere substantially with various aspects of physical, mental, and social well being [5] and many studies showed changes in functional status (i.e., mobility limitation, disability, and poor fitness level) in patients with sHT [6, 7].

On the other hand, the evidence for improvement of psychiatric symptoms with hormonal treatment (levothyroxine) of OH and the use of triiodothyronine (T3) to potentiate the response to treatment of depressive disorders suggests a direct relationship between thyroid hormones and psychiatric symptoms [8, 9]. Neurobiological evidence seems to corroborate the hypothesis of an organic basis of the effects of thyroid hormone on the brain and on psychiatric symptoms [9].

Interventions for sHT have included pharmacologic agents (i.e., hormonal treatment), psychotherapy, alternative therapies, and physical activity which can improve cardiovascular health, psychiatric symptoms, and health-related quality of life (HRQoL) [10, 11]. To date, few studies on HRQoL in subjects with sHT in response to exercise program have been reported [12, 13].

It has been proved that regular exercise positively affects the mechanisms of action associated with the physiologic deterioration and transition from subclinical thyroid disease [14]. Several authors recommended exercise to be performed within an intensity range of 40–85% of maximum oxygen consumption by VO_2max⁡_. Nevertheless, more recent studies emphasize the necessity of exploring the effects of intensity [15, 16]. Clinical thyroid disease is associated with changes in the cardiovascular system, including changes in heart rate during exercise. Considering this, we hypothesized that medium-impact exercise program would provide a more adequate exercise stimulus for improving a number of metabolic factors in females at risk for thyroid disease. Therefore, we hypothesized that a medium-impact exercise program can also improve HRQoL and cardiorespiratory fitness in females with sHT.

## 2. Materials and Methods

### 2.1. Subjects

From January 2012 to September 2012, 17 sedentary Colombian women with sHT (physical exercise less than once a week) were referred to our hospital for health examination (Servicio Medico Universidad del Valle). Subjects studied were between 40 and 65 years of age, mean age 43.1 ± 9.7 years. All patients with sHT were newly diagnosed and were positive for both antithyroid peroxidase (TPO-Ab) and antithyroglobulin (Tg-Ab) antibodies. The diagnosis of sHT was established on the basis of the elevated TSH levels and normal fT3 and fT4 values. In patients with sHT, laboratory tests were performed 1–3 days before and 12 weeks after the initiation of the training program. Obese subjects (body mass index (BMI) > 30 kg·m^−2^), smokers, and individuals with hypertension, clinical detectable coronary artery disease, and other diseases were excluded from the study. None of the patients were taking any medicine, such as estrogen supplements, T4, diuretics, antihypertensive, or hypolipidemic drugs. The University of Valle Research Ethics Committee approved this study. Informed consent was gained from all participants before the data collection began.

### 2.2. Medium-Impact Exercise Program

Preparatory training phase (weeks 1–6): the present study began with a 6-week preparatory phase of training to bring all participants up their 12 kcal/kg/wk goal. To accomplish this, all participants began their exercise program at a selected intensity set at a heart corresponding to 40–55% of VO_2max⁡_ and a frequency of 3 times per week.

Implementation of medium-impact exercise program (weeks 7–12): exercise prescription was standardized to body weight, and it was estimated that 180 minutes per week of moderate intensity exercise was equivalent to 10 to 12 kcal/kg of body weight per week. Exercise intensity was defined between 55% and 80% of VO_2max⁡_. An aerobic dose of 12 kcal/kg per week was selected for the aerobic group. Participants were weighed weekly to calculate their kcal/kg per week target. American College of Sports Medicine equations (ACSM) were used to estimate caloric expenditure rate and time required per session [17]. The exercise prescription used was established from the participants baseline exercise test and corresponded to a speed and grade associated with an upper intensity working level of 50% to 80% VO_2max⁡_ followed by recovery level of 30% VO_2max⁡_. Each session included a 30 min aerobic circuit training guided by an audio recording (tropical and latin music). The entire workout lasted about 30 minutes that, depending on the number of exercise (10 stations), were usually repeated three times. During 15 and 30 minutes throughout the routine, participants were instructed to check their pulse to ensure that they were working within their target heart rate range. Each session was preceded and followed by a gradual warm-up and cool down period, both of 10 minutes duration and consisting of walking and light, static stretching (avoiding muscle pain) of most muscle groups (upper and lower limbs, neck, and trunk muscles). The cool-down period also included relaxation and stretching exercises. Resistance exercises were performed through the full range of motion normally associated with correct technique for each exercise and engaged the major muscle groups (abdominal, dorsal, shoulder, and upper and lower limb muscles). They included 5 exercise group circuit training (50 repetitions of each) using barbells (1–3 kg/exercise) or low-to-medium resistance bands (therabands and balls). Each type of exercise on the back was performed for 2 min.

Adherence to the exercise program was encouraged by the exercise trainer and the physician who supervised each of the group sessions. Trainers were physical educators with experience in developing and monitoring exercise programs among clinical populations. In order to maximise adherence to the training program, exercise classes consisted of relevant activities for the group, 3–5 participants were accompanied by music dancing and performed in a spacious, air-conditioned room (08:00 am). Each participant met with the study dietician for nutrition assessment and counselling, and an individualized nutrition intervention plan was developed from the baseline food intake assessment, participant preferences, and the meal plan [18].

### 2.3. Health-Related Quality of Life Assessment

The Colombian standard version of the Medical Outcome Study Short-Form Health Survey (SF-12 version 2) is a questionnaire comprising 12 questions grouped into eight different domains of health: physical functioning, role limitation due to physical problems, bodily pain, general health perception, vitality, social function, role limitation due to emotional problems, and mental health. These eight scales are further clustered into the physical component summary (comprising physical function, role-physical, bodily pain and general health) and mental component summary (comprising vitality, social function, role-emotional, and mental health) [19]. The quantification of the mentioned dimensions is values varying from 0 to 100, where 0 corresponds to “worse health” and 100 to “better health.”

### 2.4. Cardiorespiratory Fitness Evaluation

Submaximal oxygen consumption (VO_2max⁡_) was assessed with the Rockport walking test. During the test, heart rate was monitored electronically using a Polar A-5 pulse meter (Polar Electro Oy, Kempele, Finland). The cardiorespiratory fitness was calculated by VO_2max⁡_ via the ACSM equation [17]:

(1) VO2max⁡(mL·Kg·min⁡−1)  =132.853−0.0769×weight−0.3877   ×height+6.315×0−3.2649   ×time−1565×heart  rate.

*Statistical Methods*. It was sought to detect a between-group difference in the change of the SF-12v2 score of 4 points (mental component summary) as it was considered clinically important. Assuming that the standard deviation in this score would be 6, similar to what was observed in a similar sample of patients [20], a total sample size of 15 would provide 80% power to detect a difference of 4 points as statistically significant. The normality of the distribution of scores was confirmed with the *Kolmogorov-Smirnov* test. The paired *t*-test was later used to estimate the difference in each outcome. The significance level was set at *P* < 0.05. All analyses were carried out by using the statistical package SPSS 16 (Chicago, IL, USA).

## 3. Results 

The mean and standard deviation of patients' age was 43.10 ± 9.70. All subjects were nonsmokers and had a sedentary lifestyle. 90% of patients were females and in the reproductive age group. The anthropometric, cardiorespiratory fitness, and metabolic profile data are listed in Table 1.

After the 12-week intervention, the participants that performed of a medium-impact exercise program showed improvements in HRQoL in most domains, particularly the vitality domain by 7 points (95% CI 2 to 11), the social functioning domain by 10 points (95% CI 4 to 15), the mental health domain by 7 points (95% CI 1 to 12), and mental component summary by 7 points (95% CI 2 to 11). One of the four domains within the physical component summary (general health domain) showed significant effect of the exercise intervention: 6 points (95% CI 1 to 11), Table 2.

**Figure 1:**
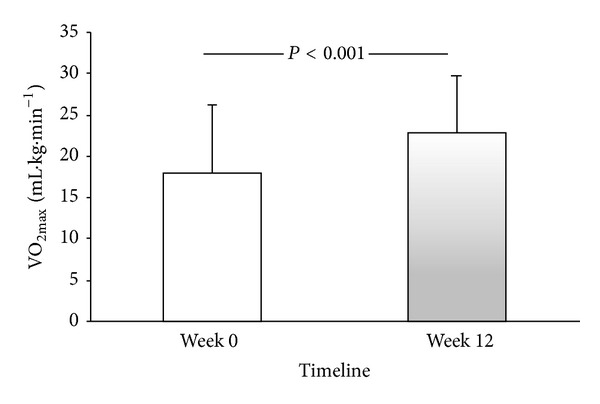
Influence of a medium-impact exercise program on cardiorespiratory fitness by submaximal VO_2max⁡_.

The paired *t*-test analysis revealed that the participants had a greater cardiorespiratory fitness at the end of the intervention, measured by Rockport walk test (*P* = 0.01) and by the submaximal VO_2max⁡_ (28%; *P* < 0.001), Figure 1.

Finally, the subjects participated 28.9 out of 36 (SD 3.2) sessions over the 12 weeks. No adverse events occurred during or after the exercise in any participant.

## 4. Discussion

The purpose of this study was to examine the influence of a medium-impact exercise program on HRQoL and cardiorespiratory fitness in females with sHT. To the authors' knowledge, this is the first systematic study evaluating the potential effectiveness of exercise program in sHT on HRQoL and VO_2max⁡_. In our clinical experience, we consider that an improvement of 4 points on the SF-12v2 resulting from this intervention is clinically important. However, no threshold has been established empirically for the amount of improvement in the SF-12v2 score that women typically feel makes aerobic training worthwhile. Our estimation of the average effect of the training had some uncertainty, with a 95% CI ranging from 1 to 10 points. Therefore, even if 4 points is a valid estimate for the smallest worthwhile effect, it must be acknowledged that it is uncertain whether the statistically significant effect of exercise is clinically worthwhile.

The median mental component summary and general health scores observed in the present study of women were similar to other studies of patient populations with conditions such as overweight [21] or sedentary [22]. There are very few studies that evaluated HRQoL in sHT [9, 10], and none included cardiorespiratory fitness and health status in the same study. It is believed that some psychological aspects of thyroid hypofunction, when present in sHT, may be influenced by physical findings, as suggested by the association between physical aspects of quality of life, in the SF-12 evaluation and cardiorespiratory fitness, specially VO_2max⁡_, in the present study. One of the primary consequences of thyroid dysfunction is lower tolerance to physical exertion, because of its implications involving the muscle and cardiovascular systems. This interferes directly with the patient's ability to perform daily activities, thereby reducing his quality of life. The study performed by Kahaly et al. [23] showed that subjects with thyroid dysfunction have reduced workload tolerance at the anaerobic threshold, compared to euthyroid subjects. According to these authors, in hyperthyroidism this exercise intolerance is caused by mitochondria oxidative dysfunction and in hypothyroidism, by inadequate cardiovascular support.

Following the 12-week exercise program, trends to improvement were seen in most domains of the HRQoL questionnaire, with statistically significant changes in the mental component summary and several of its domains. The confidence intervals were not narrow enough to confirm that the benefits would be worth the effort of exercising for these women. Nevertheless, given the other benefits of exercise in females with sHT, physicians can prescribe exercise expecting that it will improve quality of life. The recommended levels of physical activity were positively associated with one or more domains of health-related quality of life [19, 24]. In particular, physical functioning, general health, vitality, social functioning, and mental health are critically affected by the recommended level of physical activity [19]. In the current study, the physical aspects of HRQoL, such as mental component summary and general health, seemed to be more closely associated with the amount of physical activity than the physical aspects are. This finding is consistent with several previous studies [19, 20].

The intervention showed better cardiorespiratory fitness results similar to those previously reported in healthy women with high levels of physical activity [3, 6, 7]. Interestingly, it was observed that the VO_2max⁡_ diminishes progressively during thyroid hormone deficiency. So one might speculate that the increase of VO_2max⁡_ observed in the training group could have a beneficial effect in patients with thyroid disease occuring as a result of metabolic status.

The main limitation of this study is its uncontrolled design. This study, however, was conceived as preliminary research aiming to evaluate the potential usefulness of exercise in patients with sHT. Nevertheless, the finding that sHT mental symptoms were those showing greater improvement is a fact that argues against a possible placebo effect despite the lack of a control group. Investigation of other intervention components, such as behavior therapy, is also needed. In addition, future randomized controlled trials should study the effects of exercise in patients with disorders secondary to thyroid function variations and their implication, as well as therapeutic options for this highly prevalent disease.

In summary, a supervised 12-week program of primarily medium-impact exercise in females with sHT improves health-related quality of life. Moreover, this exercise program proved to have a positive influence on the functional capacity of the subjects, being effective in improving cardiorespiratory fitness.

## Figures and Tables

**Table 1:** Baseline characteristics in females with subclinical hypothyroidism.

Variable	Mean (SD)
Age (years)	43.1 (9.7)
Weight (kg)	73.57 (13.54)
BMI (kg/m^2^)	29.99 (4.48)
Waist circumference (cm)	93.47 (10.39)
Hip circumference (cm)	109.65 (10.51)
Systolic BP (mm Hg)	109.36 (8.74)
Diastolic BP (mm Hg)	66.68 (6.98)
Submaximal oxygen consumption by VO_2max⁡_	17.85 (8.40)
Maximum heart rate (beats/min)	151.56 (16.48)
Total cholesterol (mg/dL)	218.88 (25.55)
Triglycerides (mg/dL)	137.4 (64.4)
HDL-C (mg/dL)	57.18 (13.97)
LDL-C (mg/dL)	134.24 (23.26)
Glucose (mg/dL)	84.65 (11.68)
TSH (mIU/L)	2.90 (1.56)

**Table 2:** Influence of a medium-impact exercise program on health-related quality of life (SF-12v2).

	Timeline mean (SD)		Difference mean (SD)	95% CI
	Week 0	Week 12		
Physical component summary (0 to 100)	43 (7)	46 (6)	3 (10)	−8 to 2
Physical function	47 (9)	48 (9)	1 (11)	−6 to 4
Role-physical	27 (4)	28 (3)	2 (4)	−4 to 1
Bodily pain	45 (12)	52 (10)	7 (17)	−15 to 1
General health	42 (8)	48 (5)	6 (11)	1 to 11
Mental component summary (0 to 100)	40 (8)	47 (7)	7 (9)	2 to 11
Vitality	57 (9)	63 (7)	7 (12)	2 to 11
Social functioning	43 (9)	54 (6)	10 (10)	4 to 15
Role-emotional	19 (5)	21 (4)	2 (6)	−5 to 1
Mental health	48 (9)	55 (9)	7 (11)	1 to 12

SF-12v2: Colombian standard version of the Medical Outcome Study Short-Form Health Survey.
